# Supplementary figures and images for: Regulatory and evolutionary adaptation of yeast to acute lethal ethanol stress
Source: PLoS One. 2020 Nov 10;15(11):e0239528. doi: 10.1371/journal.pone.0239528 (PMC7654773; doi:10.1371/journal.pone.0239528)

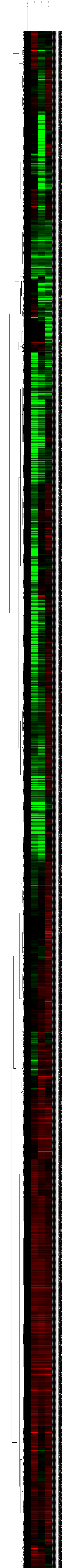

Supplement: S1 Fig — Both genes and time points were clustered, with all time points adjusted to be relative to the pre-stress time point. (TIF) [file pone.0239528.s001.tif]

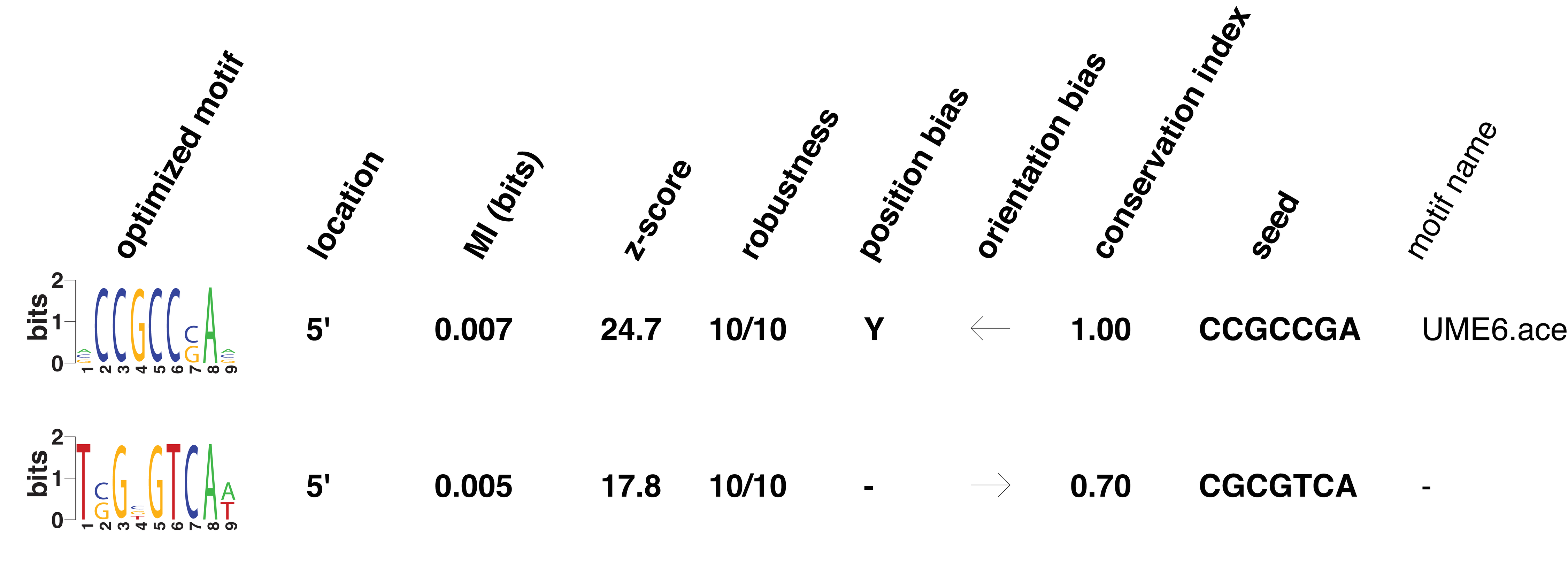

Supplement: S2 Fig — Using FIRE de novo motif discovery on condensed chromosome and spore wall assembly genes, two overrepresented motifs in the promoter region of those genes were discovered. (TIF) [file pone.0239528.s002.tif]

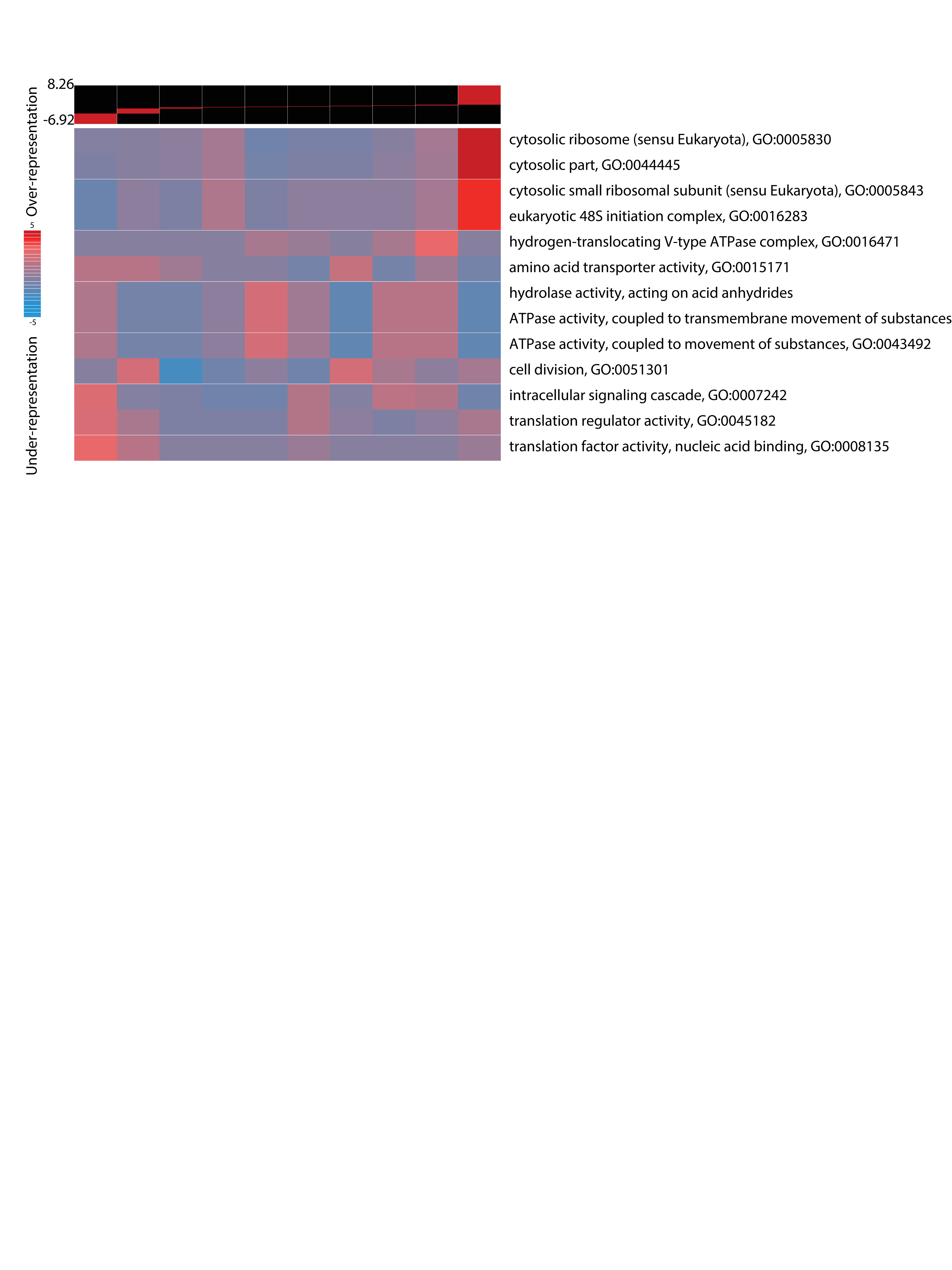

Supplement: S3 Fig — The pathways that are over- or underrepresented in ethanol stress. Overrepresented pathways are shown in red and underrepresented pathways are shown in blue. (TIF) [file pone.0239528.s003.tif]

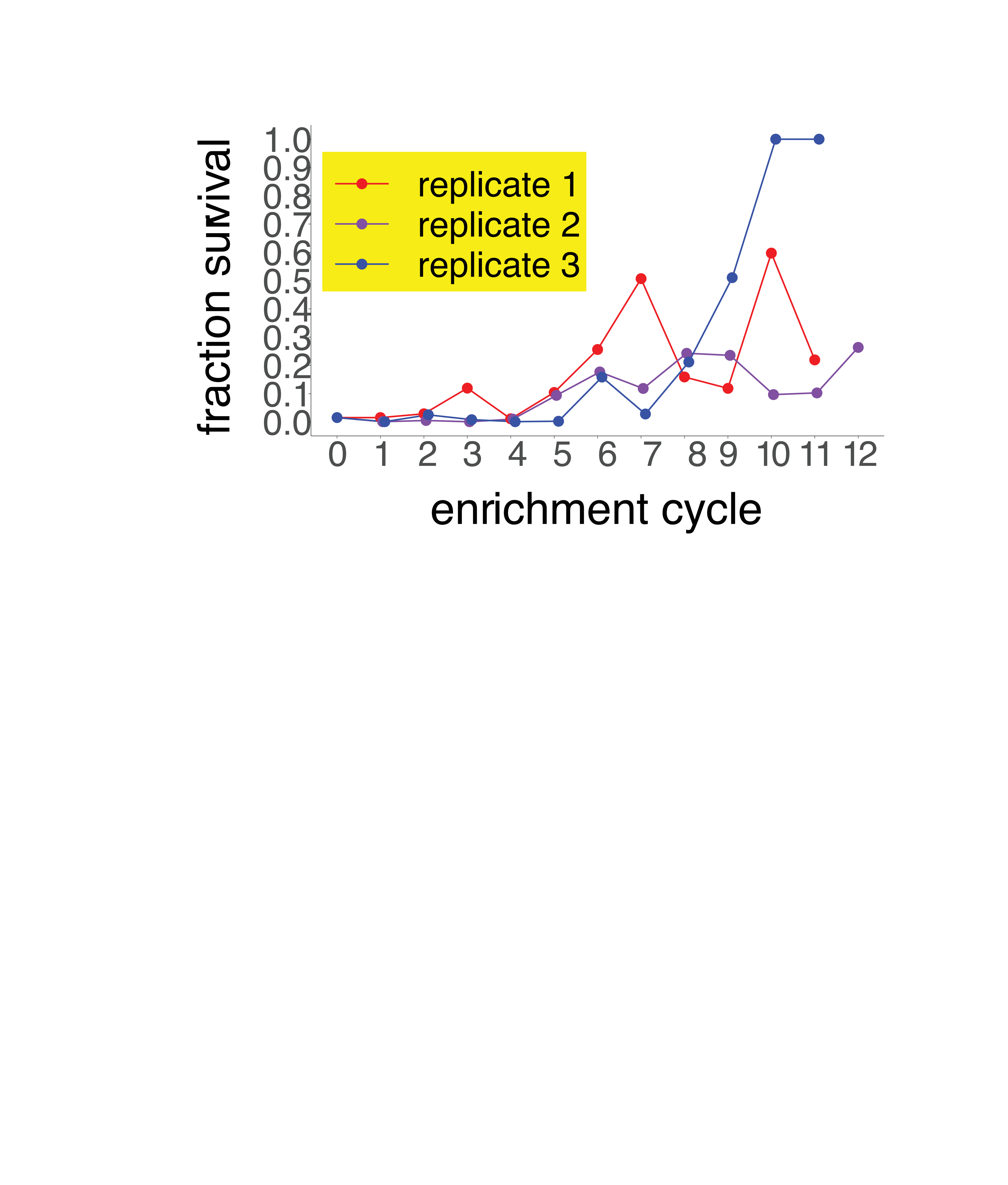

Supplement: S4 Fig — The fraction survival was calculated at each round of laboratory evolution for all three replicate lines of the wild-type background. (TIF) [file pone.0239528.s004.tif]

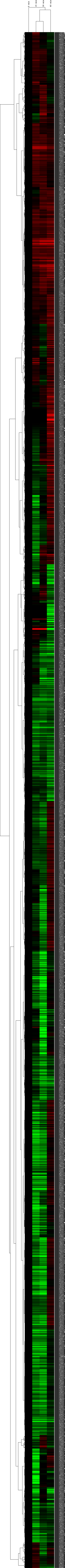

Supplement: S5 Fig — Both genes and time points were clustered, with all time points adjusted to be relative to the pre-stress time point. (TIF) [file pone.0239528.s005.tif]

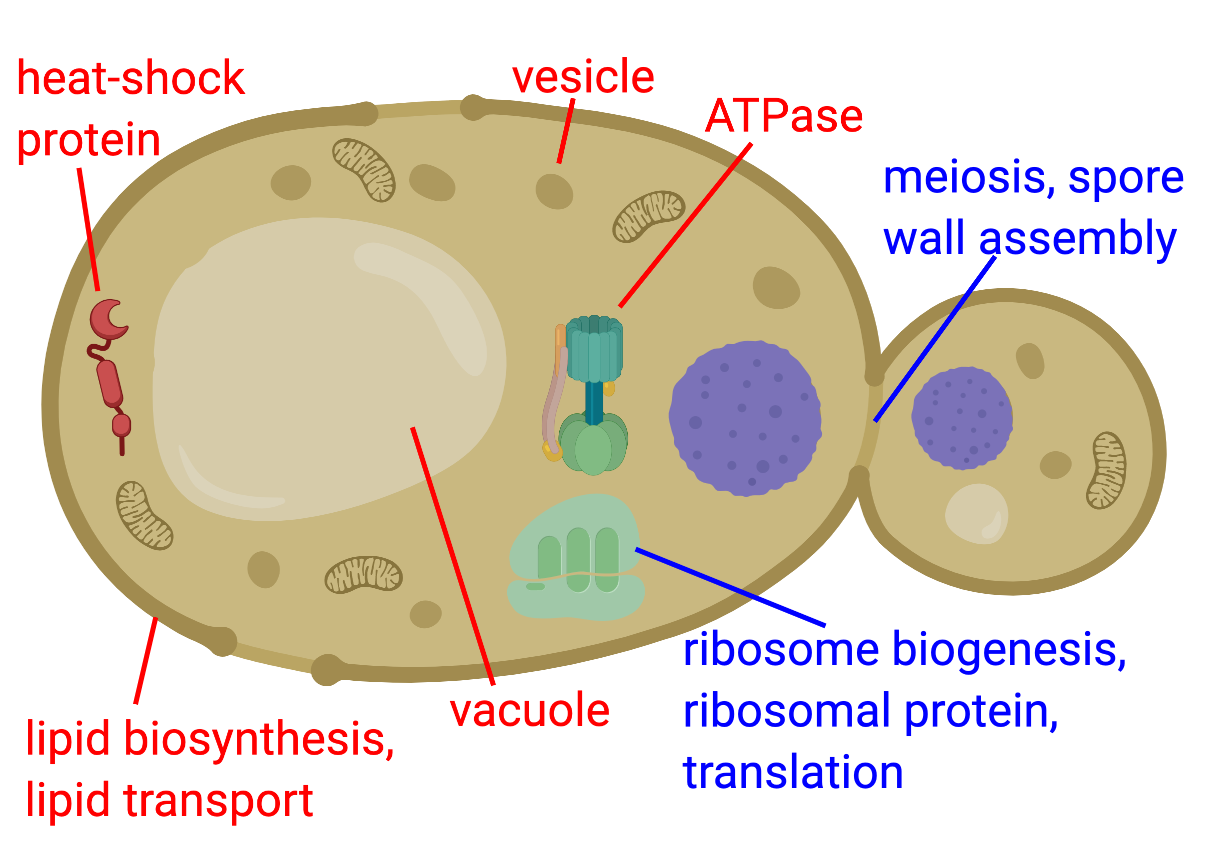

Supplement: S6 Fig — Categories in red are those significantly upregulated post-stress. Categories in blue are those significantly downregulated post-stress. (TIF) [file pone.0239528.s006.tif]
