# Supplementary material for: Regulatory and evolutionary adaptation of yeast to acute lethal ethanol stress
Source: PLoS One. 2020 Nov 10;15(11):e0239528. doi: 10.1371/journal.pone.0239528 (PMC7654773; doi:10.1371/journal.pone.0239528)
Supplement: Attachment — Submitted filename: Response to Reviewers.docx [file pone.0239528.s010.docx]

We thank the reviewers for their insightful and extremely helpful comments. We have addressed all their concerns below and have modified the manuscript to reflect the changes/additions. The reviewer comments are in regular text. Our responses are underlined. Changes made to the manuscript are presented as italic.

**Reviewer 1:**

**Major comments:**

**1.** “The experimental setup of brief stress exposure is clever and uncommon. At least two reasons can explain why only some cells survive while others don’t. Either (1) cells survived since they were able to mount a post-stress response that is essential for survival (as the propose in the abstract) or (2) that surviving cells expressed genes essential for survival pre-stress (as they mention in the introduction). The authors conclude that the post-response transcriptional response is adaptive (increases fitness) since they see a negative correlation between transcriptional change and fitness of a strain deleted for the same gene (figure 2). I am not convinced that this observation is sufficient to draw this strong conclusion. It could be that these genes are essential for ethanol survival since their basal expression prior to the stress is key for survival (and their transcriptional changes post stress is inconsequential but still correlated with survival). This conclusion can only be supported with additional experiments that will test if stress survival is diminished or is left unchanged if transcription post-stress is inhibited (of specific genes or gene sets of interest). Indeed, results from the evolved strain support the notion that the transcriptional program prior to the stress can increase stress survival. The question of whether the post-stress response is adaptive is a truly hard one and could therefore be left unanswered in the scope of this paper. However, I suggest it would be discussed in more detail in the discussion section.”

**Response:** This is an important point raised by the reviewer. Indeed, the transcriptional change post-stress is suggestive of a fitness benefit. However, in order for us to conclusively demonstrate this, additional experiments need to be performed to precisely test this hypothesis for every gene. We have therefore softened this interpretation in the main text and have added the following to the discussion section:

*“The question of whether the post-stress transcriptional response in wild-type cells is adaptive is a difficult one to conclusively answer. We believe that it does confer a fitness benefit for a couple of reasons. First, our pooled yeast deletion library experiments showed a significant negative association between transcriptional change and fitness, as shown in Fig 2C-D. Second, as shown in Table 1, the post-stress upregulation of lipid biosynthesis, ATPases, lipid transport, and vacuolar functions, all of which are normally disrupted by ethanol, suggests that the cells have mounted a response to counteract the damaging effects of ethanol. However, in order for us to conclusively demonstrate that our post-stress cells are adaptive, additional experiments would need to be performed to test individual genes of interest.”*

**2.** “A significant part of the pathway enrichment analysis from the RNA-seq dataset is built on functional enrichment (iPAGE) of individual genes sets found by k-means clustering (with 10 clusters). The choice of 10-cluster seems arbitrary (despite the short discussion in the methods section). In fact, many of these clusters seem highly correlated in the overall transcriptional response and therefore the functional enrichment done on individual clusters may not capture the underlying biology correctly. I suggest that the authors also provide a more straightforward analysis of the RNAseq dataset by performing hierarchical clustering. They can then extract the clusters from this analysis (for downstream iPAGE work). Accompanying this analysis with a figure showing the heatmap and dendrogram will allow readers to get an impartial representation of transcriptional changes at a single gene resolution. This analysis can be added to figures 2 and 4 as complementary subplots or it could be used to replace 1D and 4B.”

**Response:** We had originally performed a range of partitional and hierarchical clustering analyses on these expression data (including varying the number of clusters) and had concluded that the current presentation of 10 clusters best captures the broad dynamics observed. However, we agree with the reviewer that adding a hierarchical clustering of these data would be a very useful representation. We have, thus, performed these analyses and have provided them in the Supplementary Figures S1 Fig and S5 Fig for both the parental and evolved strains. Additionally, we have added additional text describing our observation from the hierarchical clustering.

**3.** “Overall the figures would benefit from more specific labels and titles, and consistent scales. For example, figure 3 displays survival fraction in three different ways (horizontal lines and dots for clones in 3B, mean and errorbars in 3C, no errorbar in 3D, and bars with errorbars in 3E and 3F). Figure legends are also typically too short and sometimes omit key information (for example, what do the errorbars in figure 3E and 3F represent. It would also be very helpful to mark strain JY304 on its corresponding point in figure 3B and clearly indicate in the figure labels of 3C-F that the measurements are for evolved JY304 (rather than just “evolved”).”

**Response:** We thank the reviewer for these helpful suggestions. The figures and figure legends have been updated to reflect these.

**Minor comments:**

**1.** “On page 6 top paragraph, it states transcription was monitored for 4 hours but data shared only goes to 1 hour.”

**Response:** We thank the reviewer for pointing out this error. We have corrected the text from “four hours” to “one hour”.

**2.** “There are multiple instances that give relative amounts instead of actual numbers. For example: 85% of LOF genes exhibit anti correlation (pg 12), ESR has 2x downregulated genes and this work showed 5x downregulated genes from lethal exposure. To show relativity is scalable (pg 18), ‘Many’ early stage meiosis genes poses the URS1 motif (pg 18).”

**Response:** We thank the reviewer for this suggestion. We have added absolute numbers to relevant places in the paper, including the ones mentioned on pages 12 and 18.

**3.** “On page 10 the author provides a list of references (36-41). It is unclear what information is taken from these references. Is it the gene pathway affiliation?”

**Response:** Yes, these are references to the genes/pathways. We have now split these citations to more clearly link them with their corresponding genes/pathways.

**4.** “Are the qualifications for significantly differentiated genes in previous ESR works and this work levels the same? If not, twice as many downregulated than upregulated genes in ESR as to 5x as many down regulated in these findings is not comparable.”

**Response:** Yes, in references 4 and 5, the original ESR papers, both groups used two-fold gene expression cutoffs for downregulated and upregulated genes, so we used the same criteria for our paper.

**5.** “The emphasis on these findings’ impact on industry is unclear since the acute stress used for this study is likely irrelevant for industrial ethanol production. Comments on this connection (in both the abstract and discussion) should either be further explained and supported or can be omitted altogether (the work is interesting and valuable on its own).”

**Response:** We appreciate this point raised by the reviewer and have omitted the findings’ impact on industry in both the abstract and discussion.

**Reviewer 2:**

**1.** “On the top of page 6 it says that monitoring the transcriptional response proceeding for four hours after the stress, but the post-stress RNA-seq timepoints are only 15, 30, and 60 minutes.”

**Response:** We thank the reviewer for pointing out this error. We have corrected the text from “four hours” to “one hour”.

**2.** “The main comparison of pre- and post-stress transcriptional response is done at 20% ethanol when survival looks to be near 100%. On what basis should we assume that the transcriptional response after 20% ethanol stress is the same as one after 23% or 24.5% ethanol stress, even for cells that don't die? This may in fact be true, and it makes sense, as the authors say, to avoid confounding the stress response with transcriptional changes due to death, but it isn't obvious that these transcriptional responses should necessarily be the same as the ones for cells that survive 24.5% ethanol (like the barseq and experimental evolution experiments).”

**Response:** We appreciate the reviewer’s perspective here, and we agree that there may be some differences in the transcriptional responses between 20% and 23-24% ethanol stress. However, we expect the core responses to be very similar. We have added the following to the discussion in order to point out this assumption:

*“When comparing the response to ethanol at 20% (transcriptional profiling of wild-type cells) versus the response at 24.5% ethanol (fitness profiling of deletion library), we are making the assumption that the core response to ethanol stress is similar across the regime spanning 20-24.5% ethanol. As shown by the top section of S2 Table, the pathways affected by mild ethanol stress versus acute lethal ethanol stress is very much in agreement, and we believe this core response extends into the realm of lethality. For example, downregulation of ribosomal proteins and translation are universal responses at various ethanol concentrations below, at, and above 20% ethanol. We do acknowledge, however, that there may be some differences in the transcriptional responses between 20% and 24.5% ethanol. Our analysis therefore attempts to capture the most significant global similarities and differences.”*

**3.** “The word "correlation" is used several times in the text, but actual correlations aren't run (and would be inappropriate for the data in Fig. 2C since this is a decidedly non-linear pattern). How about the more general term "association"?”

**Response:** We agree and have changed “correlation” to “association” or “relationship” in instances where an actual correlation was not determined.

**4.** “Is DeSeq2 appropriate for barseq analysis? It wasn't designed for this and so using it in this way needs some justification.”

**Response:** We added the following justification in our methods as to why we chose DESeq2:

*“We chose to use DESeq2 based on our data fitting a negative binomial distribution. Robinson et al., 2014 (reference 62) demonstrated that approaches based on negative binomial models, such as DESeq, that were originally used to analyze RNA-seq data, were directly applicable to Bar-seq data.”*

**5.** “Was DeSeq2 also used for the expression analysis? The statistical (pre-clustering) analysis of RNA-seq data wasn't described so far as I can see unless this is dealt with in the downstream programs like iPAGE.”

**Response:** DESeq2 was not used for the expression analysis. After mapping the RNA-seq reads, we converted the counts for each gene into transcripts per million, and removed any genes in which all time points had a count less than 10 (Methods section “Mapping of sequencing reads”). Before clustering, we removed all genes in which the coefficient of variation was below 0.2 (Methods section “Clustering of RNA-seq data and functional category analysis”). We did not filter the results further based on any statistical analysis because we wanted the input for clustering and iPAGE to be as unbiased as possible.

**6.** “As the authors say, the lack of a tradeoff between growth and survival is unexpected and contrary to previous findings. If there is no tradeoff then why wouldn't yeast be in this hyper-resistant pre-stress state all the time? After all, it conveys general stress protection. While I don't think the authors need to solve this mystery in this paper, it probably deserves some more discussion. Do the identities of the genes and pathways involved give any clue as to where a tradeoff might lie? Could the authors do one more experiment where they test growth rates in minimal media? Here's a (completely) speculative hypothesis: perhaps the evolved pre-stress transcriptional state renders the cells too sensitive to stress. Under the YPD growth like the authors used, things are good and the stress response isn't triggered. But under minimal media while the wild-type strain doesn't grow as fast but still grows, the evolved strain over compensates and triggers a stress response where there really isn't one. This is (as I said) completely speculative, but perhaps a few easy growth experiments in different conditions might be able to locate where a tradeoff is or further support the surprising finding that there is none.”

**Response:** Although this lack of tradeoff between growth and survival is unexpected and contrary to previous findings, there is definitely a possibility that certain environments exist in which our evolved strains would perform worse than wild-type strains. Just as the environmental stress response conveys general stress protection, it does not explain behavior in some laboratory stresses, such as various nutrient deprivations. It is certainly possible that our evolved strain performs sub-optimally in those environments as well. Additionally, this lack of tradeoff applies to acute lethal stresses as described in our manuscript, but we have no idea how the two strains compare under stresses of long durations. How the evolved cells hold up in numerous other laboratory conditions and environments is a good subject for future studies. We have expanded our discussion section to include this point as shown below:

*“While the survival advantage of strain JY304 was not accompanied by any significant reduction in the bulk growth rate of the population, there is a possibility that certain environments exist in which strain JY304 would exhibit lower fitness than the wild-type strain. Just like the ESR conveys general stress protection, it does not explain behavior in some laboratory stresses, such as various nutrient deprivations (6). It is certainly possible that strain JY304 does not perform optimally in those environments as well. Additionally, this lack of tradeoff between growth rate and survival applies to acute lethal stresses, but we have not determined how the two strains compare under stresses of long durations. How our evolved strain holds up in numerous other laboratory conditions and environments would be an important subject for future studies.”*

**7.** “The evolution results are all based on a single strain. But it looks from the methods that other strains were genome sequenced (but not transcriptionally profiled?) Also, if the evolved strains were genome-sequenced, was there anything informative there? It just seems like a loose end. How unusual is JY304? Do the other evolved strains also show this lack of growth/survival tradeoff? Was JY304 the one population on page 26 that fit the initial criteria?”

**Response:** We had preliminary whole-genome sequencing results from some of the evolved strains but did not find any significant recurring mutations. We have, thus, removed this section of the methods since these results were not mentioned anywhere else in our paper. In addition, since our goal was to compare the evolved strain with the highest survival to the wild-type strain, we did not transcriptionally profile the other strains that showed inferior survival.

**8.** “Where it is possible, the authors should post the code/scripts they used to run the analyses and process the raw data.”

**Response:** We have deposited the README file describing our general steps has the following DOI: <https://www.protocols.io/view/yang-tavazoie-sequencing-readme-bjt5knq6>. The relevant code/scripts described in the README file are located at <https://github.com/yang-jamie/SCRIPTS>.

**9.** “How dependent are the results and conclusions on the number of clusters chosen for the cluster analyses? Do results change if you go to 6 or 15 instead of 10?”

**Response:** We had performed the clustering using a range of clusters from 5-30. The ten-cluster partition provided the best balance between the number of distinct transcriptional dynamics and minimal redundancy in the pathways that were enriched within them. We have revised the Methods section “Clustering of RNA-seq data and functional category analysis” to clarify this point.

**Reviewer 3:**

**Main comments:**

**1.** “The authors emphasized the distinction between the acute stress scheme they used in multiple places. I have two problems with respect to this. First, while it is easy to understand how the pre-stress cellular state could be important for the acute stress scheme, it’s less clear to me what they mean by “long-term expression change following the stress”. In particular, how is that different from the “canonical” ethanol stress response that the cells mount following a less acute stress scheme, as studied by the others?”

**Response:** Since the “canonical” ethanol stress response was determined at ethanol concentrations well below the threshold of lethality, we cannot assume that they are necessarily similar to the response at the threshold of lethality. Our aim therefore was to determine the similarities and differences that do exist between them.

**2.** “Second, and directly related to the above, I would love to see a direct comparison of the genes identified via the RNA-seq and deletion library screening in the acute stress with the known gene expression changes and GO categories following a more mild stress challenge, as revealed in other studies. The authors did mention one of the distinctions -- among the ESR genes, the downregulated genes show more similarity while the up-regulated ESR genes are absent among those responding to the acute stress.”

**Response:** We thank the reviewer for this suggestion. We have now added a supplemental table (S2 Table) and additional text to reflect this comparison. See below:

*“S2 Table summarizes the pathways and genes that are affected during mild ethanol stress exposure and compares the directionality of their post-stress responses to the response of our data at or above the threshold of lethality. From the top section of the table, it is clear that all pathways affected by mild stress are affected in the same direction by both our transcriptional profiling and deletion library data. On the gene level, however, only 20 out of 50 genes trend in the same direction from our RNA-seq data, and only 8 out of 50 genes agree from our deletion library data. Individual genes do not overlap significantly between mild and lethal ethanol stress. In fact, even between 8% and 11% ethanol, different genes were associated with ethanol tolerance. However, on the pathway level, there is significant agreement between the responses to mild and lethal ethanol stress.”*

**Minor comments:**

**1.** “The introduction could be more tightly connected to the main questions and findings of the paper. For example, the authors spent a paragraph talking about the ESR. It is not clear, however, what is the relationship between the ESR and their finding until I read the Discussions. If each piece of background information can have a clear connection to the main points of the paper, it will make the introduction less arbitrary and more informative.”

**Response:** We thank the reviewer for this suggestion. The discussion of ESR was included because of its dominance in the literature of yeast stress response and our subsequent comparisons throughout the paper. We have now slightly modified the introduction to make this link more tangible.

**2.** “The description of the gene pathways enriched among the DGE following acute ethanol stress feel like long and diffused. This is probably a general challenge to genome-wide characterization. I wonder if the authors can attach the most important categories to a cartoon of the cell. By connecting gene categories to particular organelles of the cell or a specific biological process, e.g. DNA-repair, meiosis, bud formation etc., it will help the readers absorb and appreciate the information.”

**Response:** We thank the reviewer for this helpful suggestion. We have now added a cartoon figure, mentioned in the discussion and designated as supplementary figure S6 Fig, as shown below:

**3.** “I would like to get the authors’ interpretation of the apparently high levels of heterogeneity in the improvement in fitness among the three replicate populations in their experimental evolution.”

**Response:** As described in our methods section “Laboratory experimental evolution for enhanced survival to lethal ethanol stress”, our experiments were designed in such a way as to select for both stress defense as well as fast growth. Since slow-growing beneficial mutations will be positively selected for during stress exposure and negatively selected for during recovery, it may explain the non-monotonic and cyclical pattern of survival throughout the laboratory evolution process. Especially at the earlier cycles of the evolution, since only 1% of the yeast cells survive, there is a large bottleneck effect after each stress event, leading to high levels of heterogeneity in each replicate population.

**4.** “What are the major damages of high concentrations of ethanol to the cells? Can we tie the DE genes’ function to those damages?”

**Response:** We thank the reviewer for this suggestion. The dominant process by which yeast cells die from high concentrations of ethanol is increased membrane fluidity and subsequent decrease in membrane integrity. Other damages caused by ethanol include inhibition of H^+^-ATPase activity, inhibition of transport processes, and disruption of vacuole morphology. Our analyses suggest that our post-stress cells have altered their gene expression levels to counteract these damaging effects caused by ethanol. We have added a new table (now Table 1) to tie in the DE genes’ functions to those damages.

**5.** “In Figure 4B, it is not clear to me whether the ten categories in the mutant vs the parent strain match each other. Another way of asking this question is, is the heatmap for the parent strain the same as in Figure 1 but just reordered or are they matched to the 10 categories identified in the evolved strain? A clarification in the legend would have been helpful.”

**Response:** We thank the reviewer for pointing out this confusion. The ten categories in the evolved and parent strain are not the same. The parent strain heatmap is unaltered from Fig 1D. We have now added this clarification to the figure caption.
